# Predictors of hospital mortality among septic ICU patients with *Acinetobacter spp.*bacteremia: a cohort study

**DOI:** 10.1186/s12879-014-0572-6

**Published:** 2014-10-30

**Authors:** Andrew F Shorr, Marya D Zilberberg, Scott T Micek, Marin H Kollef

**Affiliations:** Washington Hospital Center, 110 Irving St NW, Washington, 20010 DC USA; EviMed Research Group, LLC, Goshen, MA USA; School of Public Health and Health Sciences, University of Massachusetts, Amherst, MA USA; St. Louis College of Pharmacy, 4588 Parkview Place, St. Louis, 63110 MO USA; Barnes-Jewish Hospital, St. Louis, MO USA; Division of Pulmonary and Critical Care Medicine, Washington University School of Medicine, 660 South Euclid Avenue, Campus Box 8052, St. Louis, 63110 MO USA

**Keywords:** Aevere sepsis, Acinetobacter spp, Carbapenem resistance, Inappropriate initial antibiotic therapy, Mortality, Bacteremia

## Abstract

**Background:**

We hypothesized that among septic ICU patients with *Acinetobacter spp*. bacteremia (Ac-BSI), carbapenem-resistant *Acinetobacter spp*. (CRAc) increase risk for inappropriate initial antibiotic therapy (non-IAAT), and non-IAAT is a predictor of hospital death.

**Methods:**

We conducted a retrospective cohort study of adult septic ICU patients with Ac-BSI. Non-IAAT was defined as exposure to initially prescribed antibiotics not active against the pathogen based on in vitro susceptibility testing, and having no exposure to appropriate antimicrobial treatment within 24 hours of drawing positive culture. We compared patients who died to those who survived, and derived regression models to identify predictors of hospital mortality and of non-IAAT.

**Results:**

Out of 131 patients with Ac-BSI, 65 (49.6%) died (non-survivors, NS). NS were older (63 [51, 76] vs. 56 [45, 66] years, p = 0.014), and sicker than survivors (S): APACHE II (24 [19, 31] vs. 18 [13, 22], p < 0.001) and Charlson (5 [2, 8] vs. 3 [1, 6], p = 0.009) scores. NS were also more likely than S to require pressors (75.4% vs. 42.4%, p < 0.001) and mechanical ventilation (75.4% vs. 53.0%, p = 0.008). Both CRAc (69.2% vs. 47.0%, p = 0.010) and non-IAAT (83.1% vs. 59.1%, p = 0.002) were more frequent among NS than S. In multivariate analyses, non-IAAT emerged as an independent predictor of hospital death (risk ratio [RR] 1.42, 95% confidence interval [CI] 1.10-1.58), while CRAc was the single strongest predictor of non-IAAT (RR 2.66, 95% CI 2.43-2.72).

**Conclusions:**

Among septic ICU patients with Ac-BSI, non-IAAT predicts mortality. Carbapenem resistance appears to mediate the relationship between non-IAAT and mortality.

## Background

In its 2013 report titled “Antibiotic Resistance Threats in the United States, 2013”, the US Centers for Disease Control and Prevention rated carbapenem resistance as an urgent (among Enterobacteriaceae) or serious (among *Acinetobacter spp.*) threat [1]. Since anti-pseudomonal carbapenems often represent a last-resort option, emerging resistance to this class indeed reflects a grave concern. The growing prevalence of carbapenem-resistant pathogens makes it difficult for the clinician to choose reliably what will qualify as initially appropriate antibiotic therapy (IAAT).

Understandably, of greatest concern is carbapenem resistance among Enterobacteriaceae and *Pseudomonas aeruginosa*, as they typically comprise the dominant organisms encoding carbapenemases [2]-[5]. *Acinetobacter spp*., on the other hand, is a less frequently encountered pathogen. However, the proportion of carbapenem resistance among *Acinetobacter spp*. isolates is 2–5 times that seen in *Pseudomonas aeruginosa* and *Klebsiella spp*., and over an order of magnitude that among *E. coli*[3].

In the setting of many serious infections, one of the critical determinants of the outcome is early empiric coverage for the culprit organism [6]-[14]. Failing to administer in a timely manner an agent that is *in vitro* active against the offending pathogen(s) leads to increased mortality and morbidity. Acknowledgement of this observation has led to the practice of de-escalation where one often starts with an initially broad anti-infective (or a combination of agents) and later narrows the spectrum of coverage when culture results become available [15]. Unfortunately, for *Acinetobacter spp*. it is unclear if and how one can successfully execute this strategy, particularly in light of the fact that most isolates are resistant to last-resort treatment. Similarly, as a matter of health policy and antibiotic stewardship, it is necessary to appreciate the contribution of carbapenem resistance to the poor outcomes in infections with *Acinetobacter spp.* Gaining insight into the relationship between resistance and outcomes specifically as it relates to this pathogen may help clarify the urgency of the need for novel agents with which to treat it.

We hypothesized that in the setting of severe sepsis or septic shock due to *Acinetobacter spp*., failure to receive IAAT increased the risk of death, and that, as a corollary, carbapenem resistance is a strong determinant of exposure to non-IAAT.

## Methods

### Study design and ethical standards

We conducted a single-center retrospective cohort study from January 2002 to December 2012. Barnes-Jewish Hospital is a 1,200-bed urban academic medical center located in St. Louis, MO. The study was approved by the Washington University School of Medicine Human Studies Committee and informed consent was waived.

### Study cohort

All consecutive adult ICU patients between January 2002 and December 2012 were enrolled if 1). They had a positive blood culture for *Acinetobacter spp*., and 2). There was an International Classification of Diseases, version 9, clinical modification (ICD-9-CM) code for an acute organ dysfunction [16]. Only the first episode of sepsis was included.

### Definitions

To be included in the analysis patients had to meet criteria for severe sepsis based on discharge ICD-9-CM codes for acute organ dysfunction [16]. Septic shock was present if vasopressors (norepinephrine, dopamine, epinephrine, phenylephrine or vasopressin) were initiated within 24 hours of the blood culture collection date and time. Antimicrobial treatment was deemed IAAT if the initially prescribed antibiotic regimen was active against the identified pathogen based on *in vitro* susceptibility testing and administered within 24 hours following blood culture collection; all other regimens were classified as non-IAAT. Because the role of combination therapy in treating *Acinetobacter spp.* is not well defined, combination therapy was not a criterion for defining IAAT. We also required that antibiotics be prescribed for ≥24 hours. Prior antibiotic exposure occurred within the preceding 90 days, as did prior hospitalization, while prior bacteremia was defined within 30 days of the current episode. The bacteremia was deemed to be a healthcare-associated complication (HAC) if one of the following risk factors was identified: 1). Need for dialysis; 2). Immune suppression; 3). Prior hospitalization; 4). Prior antibiotics; 5). Current infection deemed hospital-acquired blood stream infection (HABSI; onset of infection ≥2 days after admission). Carbapenem-resistant *Acinetobacter spp*. (CRAc) was present if it was the organism that grew out in at least one blood culture specimen.

### Antimicrobial susceptibility testing

The microbiology laboratory performed antimicrobial susceptibility of the isolates using the disk diffusion method according to guidelines and breakpoints established by the Clinical Laboratory and Standards Institute (CLSI) appropriate to the year the organism was isolated.

### Data elements

Patient baseline characteristics and process of care variables were collected from the automated hospital medical record, microbiology database, and pharmacy database of Barnes-Jewish Hospital. Electronic inpatient and outpatient medical records available for all patients in the BJC Healthcare system were reviewed to determine prior antibiotic exposure. The baseline characteristics collected included: age, gender, race, past history of congestive heart failure, chronic obstructive pulmonary disease, diabetes mellitus, chronic liver disease, underlying malignancy, and end-stage renal disease requiring dialysis. The Acute Physiology and Chronic Health Evaluation (APACHE) II and Charlson comorbidity scores were calculated based on clinical data present during the twenty-four hours after the positive blood cultures were obtained [17]. This was done to accommodate patients with community-acquired and healthcare-associated community-onset infections who only had clinical data available after blood cultures were drawn. The primary outcome variable was hospital mortality. Because we were interested in understanding the contribution of CRAc to the risk of receiving non-IAAT, we examined it as a secondary endpoint in a logistic regression.

### Statistical analyses

Continuous variables were reported as means with standard deviations when distributed normally, or medians with 25th and 75th percentiles when skewed. Differences between mean values were tested via Student’s *t*-test, while those between medians were examined using the Mann–Whitney *U* test. Categorical data were summarized as proportions, and the Chi-square test or Fisher’s exact test for small samples was used to examine differences between groups. We developed several multiple logistic regression models to identify clinical risk factors associated with hospital mortality. In the mortality models, all risk factors that were significant at ≤0.20 in the univariate analyses, as well as all biologically plausible factors even if they did not reach this level of significance, were included in the corresponding multivariable analyses. All variables entered into the models were examined to assess for co-linearity, and interaction terms were tested. The most parsimonious models were derived using the backward manual elimination method, and the best-fitting model was chosen based on the c-statistic. The model’s calibration was assessed with the Hosmer-Lemeshow goodness-of-fit test. To exclude the influence of time-dependent covariates on hospital mortality, we confirmed the risk factors in a Cox proportional hazards model. Similarly, the most parsimonious model for the predictors of non-IAAT was computed and its fit was tested with the c-statistic and the Hosmer-Lemeshow goodness-of-fit test.

Because both outcomes of interest in each of the models (mortality and non-IAAT) occurred with high frequency (overall mortality 49.6%, overall non-IAAT 71.0%), the adjusted odds ratios would overestimate the magnitude of the actual risk associated with each of the independent variables examined. For this reason we corrected the risk estimate according to the method of Zhang [18], and report the corrected risk ratios.

All tests were two-tailed, and a p value <0.05 was deemed *a priori* to represent statistical significance. All calculations were done in Stata/SE, version 9 (StataCorp, College Station, TX).

## Results

One hundred and thirty-one patients with severe sepsis or septic shock due to *Acinetobacter spp*. met the inclusion criteria. Among these 76 (58.0%) were CRAc. (Table 1 lists additional drug susceptibilities stratified by carbapenem resistance). Overall hospital mortality was 49.6%. The patients’ baseline characteristics are listed in Table 2. Those who died were older and had a higher comorbidity burden, as signified by the Charlson comorbidity score, than those who survived their hospitalization. A higher proportion of those patients who died prior to discharge had dialysis (28.1% vs. 15.2%, p = 0.072), prior antibiotics (75.4% vs. 57.6%, p = 0.031) and HABSI (72.3% vs. 50.0%, p = 0.009) as risk factors for a HAC than those who were discharged alive.

**Table 1:** **Susceptibilities to additional antimicrobials stratified by carbapenem susceptibility**

Drug	Carbapenem-S				Carbapenem-NS			
	S	R	I	NR	S	R	I	NR
Cefepime	31	17	2	0	1	74	1	0
Ciprofloxacin	27	17	5	1	0	74	1	1
Gentamycin	37	10	2	1	31	34	10	1
Piperacillin-Tazobactam	22	16	9	3	1	71	3	1
Tobramycin	19	1	0	30	28	20	8	20
Trimethoprim-Sulfamethoxazole	34	13	2	1	8	67	0	1
Amikacin	3	1	0	46	27	20	5	24
Ampicillin-Sulbactam	0	0	2	48	4	16	19	37
Aztreonam	1	0	0	46	41	0	0	35
Colistin	4	0	0	46	53	0	0	23
Minocycline	2	2	0	46	20	24	8	24
Tygecycline	0	2	0	48	5	7	17	47
Doxycycline	0	2	0	48	19	19	2	36

S = susceptible, NS = non-susceptible, R = resistant, I = intermediate, NR = not reported.

**Table 2:** **Baseline characteristics**

	Died	%	Survived	%	P value
	*n = 65*	*49.62%*	*n = 66*	*50.38%*	
Age, yrs					
Mean [SD]	61.9 [17.4]		55.2 [14.7]		
Median (IQR)	63 (51, 76)		56 (45, 66)		0.014
Gender, male	32	49.23%	32	48.48%	0.932
Comorbidities					
CHF	24	36.92%	14	21.21%	0.048
COPD	23	35.38%	17	25.76%	0.232
CLD	10	15.38%	3	4.55%	0.038
DM	26	40.00%	12	18.18%	0.006
CKD	22	33.85%	10	15.15%	0.013
CA	13	20.00%	13	19.70%	0.965
Charlson					
Mean [SD]	5.3 [3.6]		3.7 [3.3]		
Median (IQR)	5 (2, 8)		3 (1, 6)		0.009
Admission source					
Home	31	47.69%	32	48.48%	0.928
Another hospital	15	23.08%	21	31.82%	0.262
NH/ECF	16	24.62%	11	16.67%	0.261
Unknown	3	4.62%	2	3.03%	0.680
HAC RFs					
Dialysis	18	28.12%	10	15.15%	0.072
Immune suppression	19	29.23%	15	22.73%	0.396
Prior hospitalization	47	72.31%	46	69.70%	0.742
Prior antibiotics	49	75.38%	38	57.58%	0.031
HABSI^a^	47	72.31%	33	50.00%	0.009

SD = standard deviation; IQR = interquartile range; CHF = congestive heart failure; COPD = chronic obstructive pulmonary disease; CLD = chronic liver disease; DM = diabetes mellitus; CKD = chronic kidney disease; CA = cancer; NH = nursing home; ECF = extended care facility; HCA = healthcare-associated; RF = risk factors.

^a^Hospital-acquired BSI defined as BSI that developed after day 2 of hospitalization.

During the hospitalization and prior to sepsis onset, patients who did not survive had a far longer pre-sepsis median hospital length of stay (LOS) (13 vs. 2 days, p = 0.002) (Table 3). All markers of severity of acute illness were higher in patients who died compared to those who survived; this included the APACHE II score, the presence of septic shock and the need for mechanical ventilation (Table 3). Urine and infected line were less likely and lung was more likely as a source of infection among non-survivors compared to survivors. In contrast to pre-sepsis LOS, post-sepsis onset median LOS was far shorter among those who did not than those who did survive their hospitalization (4.5 vs. 15 days, p < 0.001).

**Table 3:** **Infection characteristics**

	Died	%	Survived	%	P value
	*n = 65*	*49.62%*	*n = 66*	*50.38%*	
LOS prior to bacteremia, days					
Mean [SD]	17.1 [23.2]		8.2 [9.9]		
Median (IQR)	13 (1, 22)		2 (0, 14)		0.002
APACHE II					
Mean [SD]	24.4 [7.9]		18.2 [6.4]		
Median (IQR)	24 (19, 31)		18 (13, 22)		<0.001
Septic shock	49	75.38%	28	42.42%	<0.001
Mechanical ventilation	49	75.38%	35	53.03%	0.008
Polymicrobial	10	18.52%	15	31.91%	0.120
Infection source^a^					
Lung	22	36.67%	19	30.16%	0.575
Urine	8	13.33%	23	36.51%	0.003
Abdomen	12	20.00%	5	7.94%	0.048
Line	5	8.33%	8	12.70%	0.308
Unknown	13	21.67%	8	12.70%	0.425
CRAc	45	69.23%	31	46.97%	0.010
Non-IAAT	54	83.08%	39	59.09%	0.002
LOS following bacteremia onset, days					
Mean [SD]	13.0 [27.0]		19.9 [18.2]		
Median (IQR)	4.5 (2, 16.5)		15 (6, 33)		<0.001

LOS = length of stay; SD = standard deviation; IQR = interquartile range; CRAc = carbapenem-resistant *Acinetobacter spp*.; IAAT = initially appropriate antibiotic therapy.

^a^Multiple sources possible.

There were substantial differences between the two groups in terms of the likelihood of CRAc as the sepsis culprit (69.2% among non-survivors vs. 47.0%% among survivors, p = 0.010) (Table 3). Additionally, non-survivors were approximately 50% more likely to receive non-IAAT than those patients who survived their hospitalization (83.1% vs. 59.1%, p = 0.002). Notably, among those patients who harbored CRAc, the risk for being treated inappropriately was 96.1%, compared to 36.4% among those with a susceptible organism, p < 0.001.

Table 4 shows the results of a multiple logistic regression model derivation to examine the variables associated with hospital mortality in this population. In this model, receiving non-IAAT was the strongest predictor of hospital death, with the corrected risk ratio of 1.42 (95% confidence interval 1.10 to 1.58, p = 0.015). A Cox proportional hazards model, confirmed non-IAAT (hazard ratio 2.37, 95% confidence interval 1.16 to 4.85, p = 0.019) and APACHE II score (hazard ratio 1.08, 95% confidence interval 1.03 to 1.12, p < 0.001; per 1 point) as risk factors for hospital death. In an additional logistic regression model to examine factors that contribute to the inappropriate choice of therapy, CRAc as the etiology of sepsis was a strong predictor of inappropriate treatment, with the corrected risk ratio measuring 2.66 (95% confidence interval 2.43 to 2.72, p < 0.001). The only other predictor retained in the model was congestive heart failure, with the corrected risk ratio 1.89 (95% confidence interval 1.01 to 2.63, p = 0.048) (AUROC 0.884, Hosmer-Lemeshow p =0.513).

**Table 4:** Independent predictors of mortality*

	Corrected risk ratio	95% confidence interval	P value
Non-IAAT	1.418	1.099-1.583	0.015
APACHE II	1.056	1.025-1.087	<0.001
Infection source: Urine	0.402	0.155-0.870	0.018

*Factors excluded from the model for collinearity: chronic kidney disease, hemodialysis, chronic obstructive pulmonary disease, diabetes mellitus (collinear with congestive heart failure); hospital-acquired blood stream infection, LOS prior to the onset of sepsis (collinear with prior antibiotics); Charlson comorbidity score (collinear with age)’ mechanical ventilation, vasopressors (collinear with APAHCE II); carbapenem resistance (collinear with non-IAAT).

Factors included but not retained in the model at the p < 0.05: congestive heart failure, chronic liver disease, prior antibiotics, age, prior hospitalization, polymicrobial infection, infection sources urine and abdomen.

IAAT = initially appropriate antibiotic therapy.

AUROC =0.801, Hosmer-Lemeshow p = 0.406.

## Discussion

In the current cohort of patients with sepsis due to *Acinetobacter spp*., the prevalence of carbapenem resistance was high at nearly 60%. More importantly, the risk for receiving non-IAAT in this setting was extreme. Specifically, the presence of CRAc as the infectious pathogen more than doubled the risk of receiving non-IAAT compared to having a carbapanem-susceptible isolate. Additionally, despite the high baseline rate of death in patients with *Acinetobacter spp*. sepsis, failure to receive appropriate therapy further increased the risk of hospital mortality.

A vast volume of research has emphasized the importance of early appropriate antimicrobial therapy in the setting of serious infections. Indeed, it has been shown in sepsis and pneumonia that the penalty for the wrong choice of empiric treatment is a 2-4-fold increase in the risk of death [6]-[8],[10]-[14], and that escalation of treatment in response to culture results fails to mitigate this increase in risk [9]. Our findings generally confirm this relationship. However, the current study adds to these earlier analyses by focusing specifically on a pathogen that is generally only sporadically found in US ICUs. The majority of earlier studies dealing with inappropriate therapy has addressed specific disease states (e.g., pneumonia), irrespective of the pathogen, or has not attempted to measure impact of inappropriate therapy in the setting of sepsis and septic shock as a syndrome. In this vein, few earlier efforts have dealt specifically with the issue of *Acinetobacter spp*. as a cause of bacteremia, and no other to the best of our knowledge has examined severe sepsis/septic shock. Indeed, the data on whether non-IAAT in the setting of *Acinetobacter* bacteremia as a contributor to the increase in the risk of death are conflicting. A small multi-center retrospective matched cohort study from Korea reported that non-IAAT was associated with a 6-fold increase in 30-day mortality [19]. Similarly, a single-center study from Turkey reported a nearly identical risk of 30-day morality to ours in association with non-IAAT (hazard ratio 2.1, 95% CI 1.2-3.7; P = 0.007) [20]. In contrast, several other studies failed to detect this relationship, though each suffered from a small sample size of bacteremia cases and other methodologic issues [21]-[24]. A cohort study from Turkey examining 100 cases of Acinetobacter bacteremia reported that carbapenem resistance was an independent risk factor for 14-day mortality [25]. However, while inappropriate empiric treatment was associated with an increase in mortality in the univariate analysis, it was not reported to be so in a multivariate analysis. It is unclear whether it was included in such and fell out or whether it was simply not examined. In either case, since we have demonstrated strong collinearity of non-IAAT with carbapanem resistance, including both in a single regression would not be statistically desirable. Another small cohort study conducted in the US examining the relationship between carbapenem resistance and mortality in *A. baumanii* bacteremia failed to detect an association, although appropriateness of treatment was an important determinant of hospital death [26]. However, such strong collinearity between non-IAAT and CRAc as we have detected in our study suggests that they may exist in the same causal pathway vis-à-vis outcomes. For this reason, it may be more statistically valid to examine them separately, as we have done, rather than in the same model.

Importantly, none of the above studies focused on the syndrome of sepsis. Because of the populations addressed in and limitations of earlier reports, ours expands upon past research exploring the mortality burden specifically related to *Acinetobacter spp*. severe sepsis and septic shock. Significantly, we illustrate that although outcomes are generally poor in persons infected with this organism, the additional impact of inappropriate therapy is substantial. As such, this suggests that there is an urgent need for agents that can provide empiric coverage for this pathogen. This last point is even more crucial in that the prevalence of drug-resistant *Acinetobacter spp*. is increasing both in the US and across the globe [27]. Thus, what may currently be of only a limited burden may in the future become much more of an issue. Conversely, our results emphasize the importance of public policy tools that foster drug development, such as the GAIN act, as well as the Food and Drug Administration’s attention to streamlining the development of antibacterial therapies in the setting of unmet medical needs [28].

Our results further suggest that carbapenem resistance is an important risk factor for receiving inappropriate empiric coverage. In other words, the key issue may not only be rapid identification of subjects specifically at risk for *Acinetobacter spp*., but may in fact be determining whether a patient is suffering from a potentially carbapenem-resistant pathogen. This not only highlights the urgent need for concerted efforts at preventing individual infections and curtailing the development and spread of resistant organisms, but also underscores the needs for novel rapid diagnostic tools. Moreover, these new diagnostics must provide clinicians up front with information about a pathogen’s likely susceptibilities rather than just simply identifying the organism.

Our study has a number of limitations. As a retrospective cohort it is prone to several forms of bias, most notably selection bias. We attempted to mitigate this by enrolling consecutive patients fitting the pre-determined enrollment criteria. Although we dealt with confounders by adjusting for those that were available, it is possible that some residual confounding remains, particularly confounding by indication. The fact that this is a single-center study in a very specific population of patients (those with *Acinetobacter spp*. sepsis) may diminish the generalizability of our results to other centers and populations. An additional potential threat to the generalizability of the findings is the fact that non-susceptibility to carbapanems was based on the corresponding year’s CLSI threshold for resistance. Applying higher MIC cut-offs either for carbapenems or other agents that have shown at least some *in vitro* susceptibilities at higher MICs would have reclassified some of the non-IAAT patients into the IAAT category [29],[30]. This reclassification would have the potential either to strengthen or to weaken the association between non-IAAT and mortality. It is important to note that, although our results strongly suggest that the association of carbapenem resistance with an increased risk of death is mechanistically related to the risk of receiving inappropriate empiric therapy, we cannot rule out that resistant *Acinetobacter spp*. may exert its lethal effect directly by virtue of higher virulence, as has been noted with other pathogens exhibiting higher MICs to certain antimicrobials [31],[32]. Because we examined hospital mortality rather than the more standard 28-day mortality as the primary outcome for our study, we may have underestimated the magnitude of this outcome.

## Conclusions

In summary, our study sheds light on the mechanism for the detrimental effect on mortality of exposure to non-IAAT in the setting of *Acinetobacter spp*. sepsis. Namely, it is the pathogen’s resistance to carbapenems, the class of last resort, that appears to mediate at least in part this adverse outcome. In this way, this pathogen is an eloquent illustration of a recent World Health Organization’s statement: “A post-antibiotic era – in which common infections and minor injuries can kill – far from being an apocalyptic fantasy, is instead a very real possibility for the 21st Century” [33].

## Authors’ contributions

MDZ participated in conception, design, analysis and interpretation of the data, drafted the manuscript and has given final approval for the version to be published. MDZ takes responsibility for data accuracy and analytic and reporting integrity of the study. AFS participated in conception, design, analysis and interpretation of the data. He was involved in revising the manuscript critically for important intellectual content, and has given final approval for the version to be published. STM participated in conception, design, acquisition and interpretation of the data. He was involved in revising the manuscript critically for important intellectual content, and has given final approval for the version to be published. MHK participated in conception, design, acquisition and interpretation of the data. He was involved in revising the manuscript critically for important intellectual content, and has given final approval for the version to be published. All authors read and approved the final manuscript.
